# Novel Polycarbo-Substituted Alkyl (Thieno[3,2-*c*]quinoline)-2-Carboxylates: Synthesis and Cytotoxicity Studies

**DOI:** 10.3390/molecules191118527

**Published:** 2014-11-13

**Authors:** Malose Jack Mphahlele, Marole Maria Maluleka, Tshepiso Jan Makhafola, Peace Mabeta

**Affiliations:** 1 Department of Chemistry, College of Science, Engineering and Technology, University of South Africa, P.O. Box 392, Pretoria 0003, South Africa; E-Mail: malulmm@unisa.ac.za; 2 Department of Life and Consumer Sciences, College of Agriculture and Environmental Sciences, University of South Africa, Private Bag X06, Florida 1710, South Africa; E-Mail: makhat@unisa.ac.za; 3 Department of Anatomy and Physiology, University of Pretoria, P/Bag X04, Pretoria 0110, South Africa; E-Mail: peace.mabeta@up.ac.za

**Keywords:** 6,8-dibromo-4-chloroquinoline-3-carbaldehyde, Suzuki-Miyaura cross-coupling, alkyl thieno[3,2-*c*]quinoline-2-carboxylates, MCF-7 cell line, cytotoxicity

## Abstract

Direct one-pot base-promoted conjugate addition–elimination of 6,8-dibromo-4-chloroquinoline-3-carbaldehyde with methyl mercaptoacetate and subsequent cyclization afforded methyl [(6,8-dibromothieno[3,2-*c*]quinoline)]-2-carboxylate. The latter undergoes Suzuki-Miyaura cross-coupling with arylboronic acids to yield exclusively the corresponding alkyl [(6,8-diarylthieno[3,2-*c*]quinoline)]-2-carboxylates,. The cytotoxicity of the prepared compounds was evaluated against the human breast cancer cell line MCF-7 using the MTT assay. The effects of compounds **2**, **3c** and **4d** on cell kinetics were further determined using the xCELLigence Real Time Cell Analysis (RTCA) system. In both the MTT assay and Real Time Cell Analysis, the compounds inhibited cancer cell growth in a dose- and time-dependent manner. Furthermore, on the basis of the calculated LC_50_ values, the compounds compared favourably with nocodazole, a well-established anticancer drug.

## 1. Introduction

The synthesis of thieno[3,2-*c*]quinoline-based compounds continues to attract a great deal of attention in research because of their rich biological activities and excellent pharmacological properties. This moiety is widely distributed in biologically important compounds including antibacterial [1], anticancer [2] and anti-inflammatory agents [3]. Some thieno[3,2-*c*]quinoline derivatives have also been found to inhibit protein kinases in cancer cells [4,5]. A series of substituted 2,4-dimethyl-thieno[3,2-*c*]quinolones was previously prepared via intramolecular cyclization and subsequent aromatization of 3-(2-chloroprop-2-en-1-yl)- and 3-(2-oxopropyl)-2-methylquinolin-4-thiols [6]. The reaction of 4-chloroquinolin-2(1*H*)-ones with thioglycolic acid and/or 2-sulfanylpropionic acid in the presence of a base as catalyst and subsequent polyphosphoric acid-promoted cyclodehydration, on the other hand, yielded the 3-hydroxythieno[3,2-*c*]quinolin-4(1*H*)-ones [7]. A single-pot synthesis of thieno[3,2-*c*]quinolin-4(5*H*)-ones involving successive *m*-chloroperbenzoic acid-mediated oxidation of 4-(4'-aryloxybut-2'-ynyl)thioquinolin-2(1*H*)-ones in chloroform at 0–5 °C followed by heating and treatment with 20% KOH (aq) has also been described in the literature [8]. Likewise, thieno[3,2-*c*]-quinolinones were prepared via thio-Claisen rearrangement of the corresponding 4-allylthio-1-methylquinolin-2(1*H*)-ones in refluxing *N*,*N-*diethylaniline [9]. Moreover, a one-pot conjugate addition-elimination of the analogous 2,4-dichloroquinoline-3-carbonitrile with ethyl mercaptoacetate previously afforded ethyl 3-amino-4-chlorothieno[3,2-*c*]quinoline-2-carboxylates [4,10]. Less traditional synthesis of 2-substituted thieno[3,2-*c*]quinolines, which involves sequential amination and intramolecular palladium-catalyzed direct arylation of thiophene-3-carbaldehyde with 2-haloanilines to afford 2-arylthieno[1,3-*c*]quinolines in a single-pot operation has been reported [11]. 

New findings on the biological and photophysical properties of polycarbo-substituted quinolines and their heteroannulated derivatives reveal a need to increase the diversity of carbon-containing substituents around the heterocyclic scaffold. The quinoline derivatives bearing aryl-, alkynyl- or alkenyl groups, for example, have been found to serve as potent inhibitors of tyrosine kinase PDGF-RTK [12], anti-retroviral agents [13] or LTD_4_ receptor antagonists [14], respectively. To this end, the halogen-containing quinoline moiety has established itself as a useful scaffold for structural elaboration via metal-catalyzed cross-coupling reactions to afford novel polycarbo-substituted derivatives. Although a wide variety of thieno[3,2-*c*]quinolines have been prepared before, derivatives containing aryl, alkenyl or alkynyl groups on the fused benzo ring have not been explored thus far. This prompted us to investigate the possibility to synthesize halogenated thieno[3,2-*c*]quinoline as scaffold for palladium catalyzed Suzuki-Miyaura cross-coupling with aryl- and arylvinylboronic acids to afford novel polyaryl-substituted thieno[3,2-*c*]quinolines with potential anticancer properties.

## 2. Results and Discussion

### 2.1. Chemistry

**Scheme 1:**
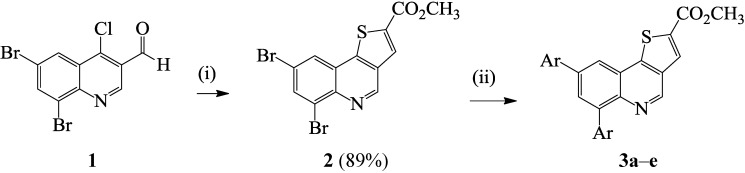
Suzuki-Miyaura cross-coupling of specially prepared **2** to afford **3a**–**e**.

**Scheme 2:**
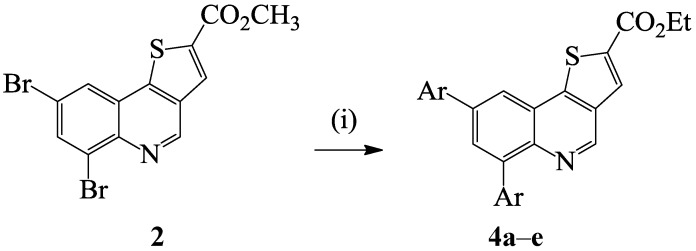
One-pot Suzuki-Miyaura cross-coupling and trans-esterification of **2** to afford **4a**–**e**.

**Scheme 3:**
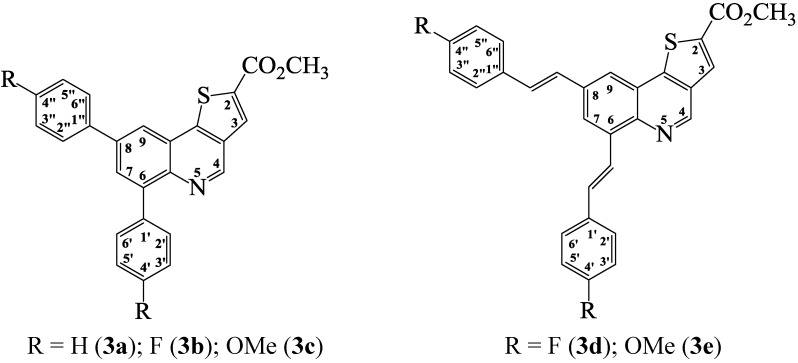
Generalized structures for the methyl thieno[3,2-*c*]quinoline-2-carboxylates **3a**–**c** and **3d**, **e**.

Although β-chlorovinylaldehydes such as the 2/4-chloroquinoline-3-carbaldehydes have been exploited extensively in the synthesis of angular and linear azoloquinolines [10,15], to our knowledge, there is no literature precedent for their transformation into thienoquinoline derivatives. Recourse to the literature, on the other hand, revealed a single-pot base-promoted conjugate addition–elimination of the 4-chloro-5,6-diphenyl-2*H*-thiopyran-3-carbaldehyde with methyl thioglycolate and subsequent heteroannulation to afford 4*H*-thieno[3,2-*c*]thiopyran [16]. Based on this literature precedent, we reacted the known 6,8-dibromo-4-chloroquinoline-3-carbaldehyde (**1**) [15] with methyl mercapto-acetate in the presence of anhydrous potassium carbonate as a base in acetonitrile under reflux (Scheme 1). We isolated by filtration a single product which was characterized using a combination of ^1^H-NMR (due to poor solubility in DMSO-*d*_6_) and IR spectroscopic techniques as well as mass spectrometry as methyl [(6,8-dibromothieno[3,2-*c*]quinoline)]-2-carboxylate **2**. We took advantage of the bromine atoms to investigate the reactivity of compound **2** in palladium catalyzed Suzuki-Miyaura cross-coupling with arylboronic and arylvinylboronic acids. We first explored the use of tetrakis(triphenylphosphine)palladium(0) and dichlorobis(triphenylphosphine)(II) as sources of the active Pd(0) species and potassium carbonate as a base in dimethyl formamide under reflux. The reaction of **2** with phenylboronic acid (1–2 equiv.) using Pd(PPh_3_)_4_ as catalyst source afforded the diarylated product in reduced yield (<25%) after 24 h along with considerable amount of the starting material. We then used PdCl_2_(PPh_3_)_2_ as catalyst source since the Pd(0) complex (Pd(0)(PPh_3_)_2_Cl^−^) generated from its reduction is known to be more than 30 times faster in the oxidative-addition step than that formed from Pd(0)(PPh_3_)_4_[17]. We isolated the disubstituted product in 50% yield after 18 h. The prolonged reaction time and reduced yield prompted us to use dichlorobis(tricyclohexyl-phosphine)palladium(II) (PdCl_2_(PCy_3_)_2_) as Pd(0) source. The choice of this catalyst source was based on the fact that alkylphosphine ligands in the Pd source coordinate with palladium and increase its electron density more so than arylphosphines and, in turn, accelerate the oxidative-addition step [18]. Complete transformation of the substrate and improved yield of the product (90%) were observed with PdCl_2_(PCy_3_)_2_ as active Pd(0) source on **2** in the presence of K_2_CO_3_ in DMF under reflux for 4 h (Scheme 2). The reaction conditions using PdCl_2_(PCy_3_)_2_ were, in turn, extended to other aryl- and arylvinylboronic acids to afford products **3a**–**e** in appreciable yields without the need for column chromatography. The highest yield was observed with phenylboronic acid whereas the highly nucleophilic 4-methoxyphenylboronic acid led to reduced yield of the cross-coupled product. Lack of selectivity is rationalized in terms of C–Br bond strengths based on computational methods at B3LYP and G3B3 levels, which previously revealed that all of the positions on the fused benzo ring of quinolines bearing identical halogen atoms have comparable C–X bond strengths [19]. Hitherto, the analogous 1-aryl-6,8-dibromo-1*H*-pyrazolo[4,3-*c*]quinolones [16], 2-aryl-6,8-dibromo-4-methoxy-quinolines [20], 5,7-dibromoquinoline [21], 8-benzyloxy-5,7-dibromoquinoline [22], and 3,6,8-tribromoquinoline [23] have also been found to undergo Suzuki-Miyaura cross-coupling without selectivity. Lack of selectivity was also observed in an attempted one-pot borylation of 8-bromo-6-chloroquinoline with bis(pinacolato)-diboron followed by Suzuki-Miyaura cross-coupling with aryl halides and the authors, in turn, used an excess of bis(pinacolato)diboron (2.2 equiv.) and phenylbromide to afford 6,8-diphenylquinoline in 94% yield [24]. Due to lack of solubility in most deuterated solvents and poor solubility in DMSO-*d*_6_, compounds **3a**–**e** were characterized mainly on the basis of ^1^H-NMR spectroscopic data and in some cases complemented with ^13^C-NMR spectral data as well as IR (thin film method) spectroscopy (see Scheme 3). The accurate calculated *m*/*z* values for compounds **3**, nevertheless, represent in each case a closest fit consistent with the assigned molecular structures.

*Reagents and conditions*: (i) HSCH_2_CO_2_Me (2.5 equiv.), K_2_CO_3_, CH_3_CN, reflux, 3 h; (ii) ArB(OH)_2_ (2.5 equiv.), PdCl_2_(PCy_3_)_2_, K_2_CO_3_, DMF, reflux, 4 h.

**Table:** 

Compound	Ar	% Yield
**3a**	-C_6_H_5_	92
**3b**	4-FC_6_H_4_-	65
**3c**	4-MeOC_6_H_4_-	63
**3d**	4-FC_6_H_4_CH=CH-	96
**3e**	4-ClC_6_H_4_CH=CH-	81

*Reagents and conditions*: (i) ArB(OH)_2_ (2.5 equiv.), PdCl_2_(PCy_3_)_2_, K_2_CO_3_, DMF-ethanol (3/1; v/v), reflux, 4 h.

**Table:** 

Compound	Ar	% Yield
**4a**	-C_6_H_5_	96
**4b**	4-FC_6_H_4_-	70
**4c**	4-ClC_6_H_4_-	71
**4d**	4-MeOC_6_H_4_-	62
**4e**	4-ClC_6_H_4_CH=CH-	67

**Scheme 4:**
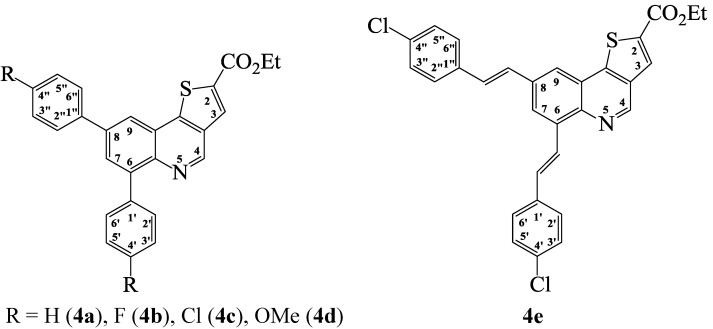
Generalized structures for the ethyl thieno[3,2-*c*]quinoline-2-carboxylates **4a**–**d** and **4e**.

With the intention of increasing the solubility of the resulting cross-coupled products, we decided to investigate suitable reaction conditions for the direct one-pot synthesis of the corresponding ethyl diaryl(thieno[3,2-*c*]quinoline)-2-carboxylate derivatives from compound **2**. Literature precedent reveals that potassium carbonate reacts with alcohols (MeOH, EtOH) at room temperature to generate alkali metal alkoxides and their bicarbonate salts [25]. We envisioned that under the same conditions used to effect the Suzuki cross-coupling of **2** with arylboronic acids, the use of DMF and ethanol as a co-solvent and potassium carbonate as a base might promote trans-esterification and C*sp*^2^–C*sp*^2^ bond formation in a single-pot operation. To confirm this assumption, we subjected compound **2** to phenylboronic acid (2.5 equiv.) and PdCl_2_(PCy_3_)_2_ as catalyst, K_2_CO_3_ as a base in DMF-ethanol mixture (3/1, v/v) under reflux for 4 h (Scheme 2). To our delight, we isolated ethyl 6,8-diphenylthieno[3,2-*c*]quinoline-2-carboxylate (**4a**). These reaction conditions were extended to the other arylboronic acid derivatives and 4-chlorophenylvinylboronic acid with **2** to afford products **4b**–**e**. These compounds were characterized using a combination of NMR, IR and mass spectroscopic techniques (see Scheme 4). Within this series of compounds, the highest yield was also observed from the cross-coupling of **2** with phenylboronic acid whereas the highly nucleophilic 4-methoxyphenylboronic acid afforded the cross-coupled product in relatively low yield.

### 2.2. Cytotoxic Activity against Human Breast Cancer Cell Line MCF-7

Breast cancer is one of the leading causes of cancer deaths among females in developing countries [26]. As a prelude to polycarbo-substituted thienoquinolines with antitumour properties, we decided to screen compounds **2**–**4** for *in vitro* cytotoxic properties against human breast cancer cell line MCF-7 using the 3-(4,5-dimethylthiazole-2-yl)-2,5-diphenyltetrazoliumbromide (MTT) protocol at different molar concentrations (0.01, 0.10, 1.0 and 10 μg/mL) with DMSO and nocodazole as the negative and positive control, respectively. The MTT assay gives an indirect measure of cytotoxicity by measuring metabolically active cells. In this assay, 3-(4,5-dimethylthiazole-2-yl)-2,5-diphenyltetrazoliumbromide becomes reduced by mitochondrial dehydrogenases in living cells to a blue-magenta coloured formazan precipitate. Cytotoxic compounds damage and destroy cells, and thus decrease the reduction of MTT to formazan [27]. The absorption of dissolved formazan in the visible region is known to correlate with the number of intact living cells. 

**Figure 1:**
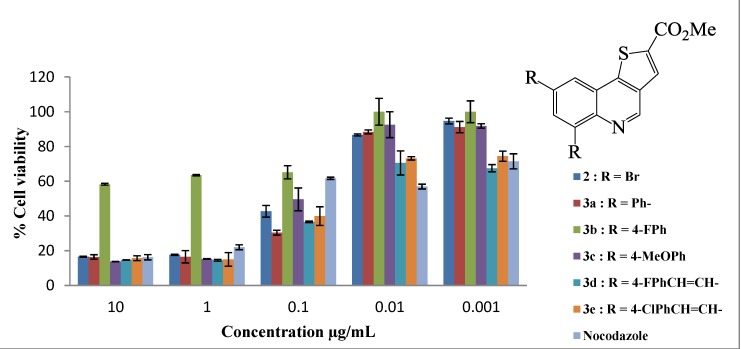
*In vitro* antiproliferative activities of thienoquinolines **2** and **3a**–**e** on human breast cancer cell line MCF-7. Results are presented as percentage cell viability (±standard deviation (SD) from three individual experiments) using the MTT assay.

**Figure 2:**
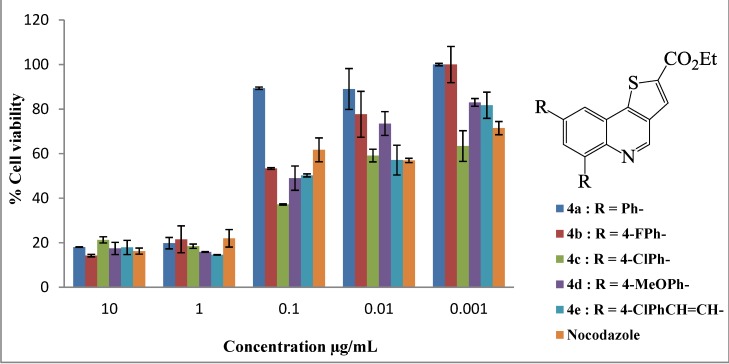
*In vitro* cytotoxic activities of thienoquinolines **4a**–**e** on human breast cancer cell line MCF-7. Results are presented as percentage cell viability using the MTT assay.

All the compounds assayed had varying degrees of toxicity and resulted in a decrease in cell viability at all the concentrations tested (Figure 1 and Figure 2). The LC_50_ values of compounds **2**–**4** are shown in Table 1. Six of the eleven compounds assayed in the MTT assay were more cytotoxic to the human breast adenocarcinoma (MCF-7) cells when compared to nocodazole which is a widely used drug in cancer therapy (*i.e.*, compounds **2**, **3c**, **3d**, **3e**, **4c** and **4e**; all with LC_50_ values less than 0.13 μg/mL). Compound **2** bearing two bromine atoms at the C-6 and C-8 positions was found to be less toxic compared to most of the polycarbo-substituted derivatives. Replacement of the two bromine atoms with the moderately resonance donating 4-fluorophenyl groups resulted in decreased cytotoxicity for the 6,8-bis(4-fluorophenyl)thienoquinoline **3b** (Figure 1). Increased cytotoxicity compared to the other aryl-substituted derivatives was, however, observed for compounds **3c** (Figure 1) and **4d** (Figure 2) bearing the strong conjugative 4-methoxyphenyl group.

**Table 1:** Cytotoxic effects of polycarbo-substituted alkyl (thieno[3,2-*c*]quinoline)-2-carboxylates against human breast adenocarcinoma cell line (MCF-7cells) MTT assay. The results are presented as LC_50_ (µg/mL) ± standard deviation (SD) from three individual experiments.

Compound	LC_50_ (µg/mL)
**2**	0.11 ± 0.01
**3a**	0.30 ± 0.03
**3b**	˃10.00 ± 0.004
**3c**	0.094 ± 0.03
**3d**	0.014 ± 0.002
**3e**	0.022 ± 0.003
**4a**	1.84 ± 0.02
**4b**	1.84 ± 0.003
**4c**	0.01 ± 0.03
**4d**	0.16 ± 0.04
**4e**	0.04 ± 0.02
Nocodazole	0.13 ± 0.009

**Figure 3:**
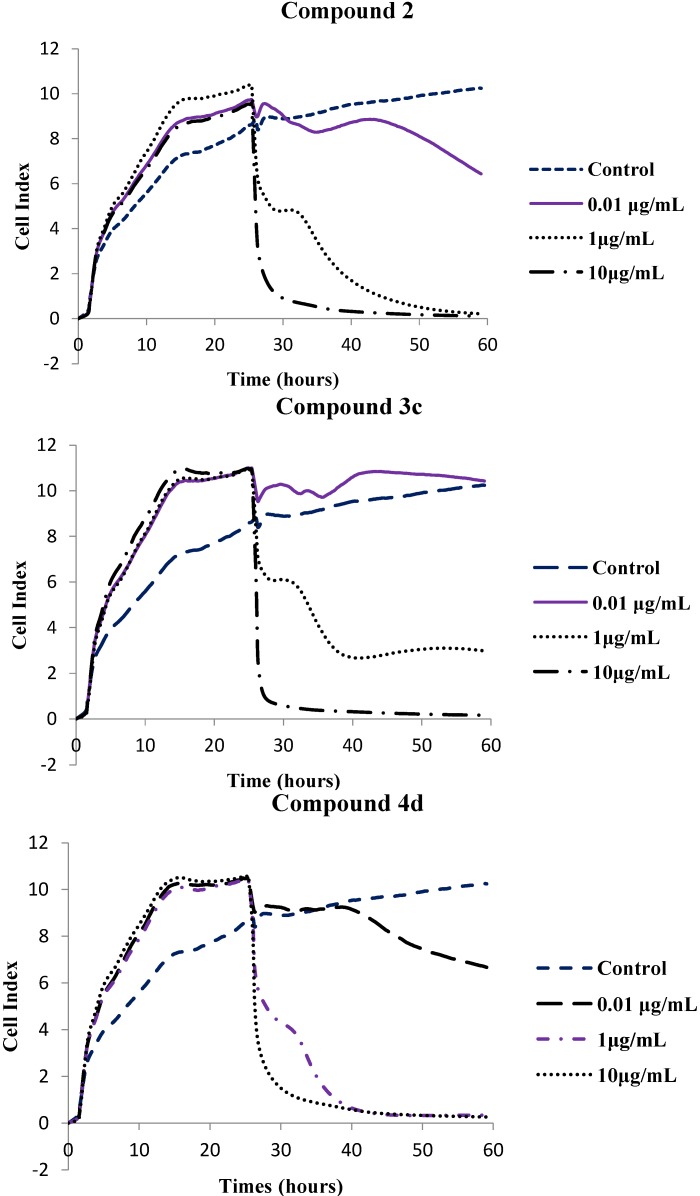

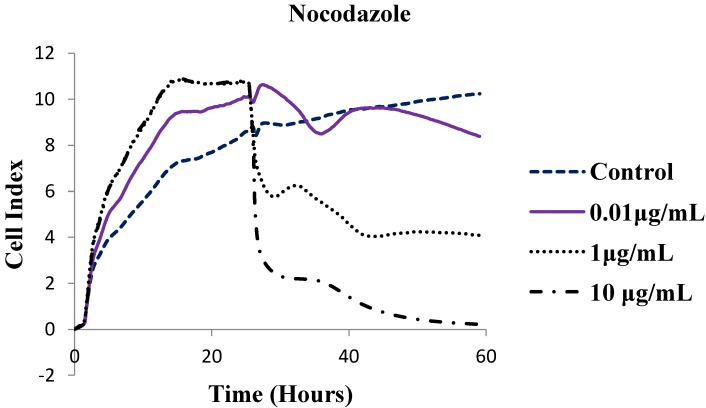
*In vitro* anti-proliferative effects of compounds **2**, **3a**, **4d** and Nocodazole on human breast cancer cells at concentrations 0.01, 1 and 10 μg/mL against 0.001% DMSO using the xCELLigence Real Time Cell Analysis System. Cell Index (CI) values are normalized to the time point of compound administration, showing a dose- and time-dependent antigrowth effect.

The cytotoxic effects of selected compounds, namely, the parent compound **2** and polycarbo-substituted thienoquinolines **3c** and **4d** were further determined using the xCELLigence Real Time Cell Analysis (RTCA) instrument for 60 h and the corresponding software was used to calculate LC_50_ values. The xCELLigence system allows for real-time monitoring of cellular phenotypic changes by measuring electrical impedance using interdigitated microelectrodes integrated into the bottom of each well of the tissue culture E-Plates 96. Impedance measurements are displayed as Cell Index (CI) values. These values provide quantitative information about the biological status of the cells including cell number, cell viability and cell morphology [28]. The compounds induced a dose- and time-dependent growth inhibitory effect as evident in Figure 3. The cell index (CI) of compounds **2**, **3c** and **4d** was found to decrease with increasing test concentrations, and time of incubation and exposure. 

All three compounds assayed using the xCELLigence RTCA system had slightly lower LC_50_ values than nocodazole (LC_50_ = 0.171 μg/mL). The parent compound (compound **2**) had an LC_50_ value of 0.167 μg/mL and the 6,8-bis(4-methoxyphenyl)–substituted derivatives **3c** and **4d** had LC_50_ values of 0.094 and 0.169 μg/mL, respectively. We rationalize the observed cytotoxicity of compounds **3c** and **4d** as a consequence of strong conjugative effect of the 4-methoxyphenyl groups that renders the quinoline ring sufficiently basic so that it is protonated at physiological pH (pK > 8.4). The slight reduction in cytotoxicity of **4d** compared to **3c** is presumably the consequence of the presence of a bulky ethoxy group. In our view, the electronic effect and lipophilicity of the substituent on the *para* position of the 6- and 8-phenyl rings seem to be critical for the cytotoxicity of these compounds. Although MTT and RTCA assay measure cell death and/or cell proliferation using different end-points, the LC_50_ values obtained from the MTT assay (Table 1) compare favorably with the LC_50_ values obtained in the xCELLigence Real Time Cell Analysis (RTCA) system (Table 2). Moreover, literature precedent has shown that there is a positive correlation between the MTT assay and RTCA assay results [29]. However, the RTCA system has been reported to be the most suitable system for studies relating to cell response to toxic agents [29]. 

**Table 2:** Cytotoxic effects of compounds **2**, **3c**, **4d** and nocodazole against human breast adenocarcinoma cell line (MCF-7 cells) using the xCELLigence Real Time Cell Analysis (RTCA) system.

Compound	IC_50_ (µg/mL)
**2**	0.167
**3c**	0.094
**4d**	0.169
Nocodazole	0.171

## 3. Experimental Section

### 3.1. General Information

Melting points were recorded on a Thermocouple digital melting point apparatus and are uncorrected. IR spectra were recorded as powders using a Bruker VERTEX 70 FT-IR Spectrometer (Bruker Optics, Billerica, MA, USA) with a diamond ATR (attenuated total reflectance) accessory by using the thin-film method. For column chromatography, Merck kieselgel 60 (0.063–0.200 mm) was used as stationary phase. NMR spectra were obtained as DMSO-*d*_6_ solutions using a Varian Mercury 300 MHz NMR spectrometer (Varian Inc., Palo Alto, CA, USA) and the chemical shifts are quoted relative to the solvent peaks. Low- and high-resolution mass spectra were recorded at the University of Stellenbosch Mass Spectrometry Unit using Synapt G2 Quadrupole Time-of-flight mass spectrometer (Waters Corp., Milford, MA, USA). The synthesis and characterization of substrate **1** have been described elsewhere [15].

#### 3.1.1. Synthesis of Methyl [(6,8-Dibromothieno[3,2-*c*]quinoline)]-2-carboxylate (**2**)

A stirred mixture of β-chlorovinylaldehyde **1** (1.37, 3.92 mmol), anhydrous K_2_CO_3_ (1.50 g, 10.87 mmol) and methyl mercaptoacetate (0.62 g, 5.88 mmol) in dry acetonitrile (50 mL) was refluxed for 3 h. The mixture was allowed to cool to room temperature and quenched with cold water. The precipitate was filtered on a sintered funnel, washed with cold water and recrystallized from toluene to afford **2** as a white solid (1.40 g, 89%), mp. 248–250 °C; ν_max_ (ATR) 747, 796, 1021, 1266, 1564, 1634, 1719 cm^−1^; δ_H_ (300 MHz, DMSO-*d*_6_) 3.95 (3H, s, -CH_3_), 8.40 (1H, d, *J* 1.2 Hz, 9-H), 8.51 (1H, s, 3-H), 8.64 (1H, d, *J* 1.2 Hz, 7-H), 9.48 (1H, s, 4-H); *m*/*z* 400 (100, MH^+^); HRMS (ES): MH^+^, found 401.8626. C_13_H_8_NO_2_S^79^Br_2_^+^ requires 401.8620.

#### 3.1.2. Typical Procedure for the Suzuki-Miyaura Cross-Coupling of **2** with Arylboronic Acids

A mixture of **2** (1 equiv.), arylboronic acid (2.5 equiv.), PdCl_2_(PCy_3_)_2_ (10% of **2**) and K_2_CO_3_ (2.5 equiv.) in DMF (5 mL/mmol of **2**) in a two-necked flask equipped with a stirrer bar, rubber septum and a condenser was flushed for 20 min with argon gas. A balloon filled with argon gas was then connected to the top of the condenser and the mixture was heated with stirring at 80–90 °C under argon atmosphere for 4 h. The mixture was allowed to cool to room temperature and then poured into an ice-cold water. The resulting precipitate was filtered on a sintered funnel and recrystallized to afford **3**. The following products were prepared in this fashion:

*Methyl 6,8-diphenylthieno[3,2-*c*]quinoline-2-carboxylate* (**3a**). Solid (0.44 g, 92%), mp. 168–170 °C (toluene); ν_max_ (ATR) 688, 750, 920, 1067, 1158, 1289, 1479, 1716 cm^−1^; δ_H_ (300 MHz, DMSO-*d*_6_) 3.94 (3H, s, -CH_3_), 7.45–7.58 (6H, m, Ar-H), 7.75 (2H, d, *J* 6.0 Hz, 2'',6''-H), 7.98 (2H, d, *J* 7.5 Hz, 2',6'-H), 8.08 (1H, d, *J* 1.8 Hz, 7-H), 8.45 (1H, s, 3-H), 8.48 (1H, d, *J* 1.8 Hz, 9-H), 9.32 (1H, s, 4-H); δ_C_ (75 MHz, DMSO-*d*_6_) 53.6, 121.0, 124.2, 127.8, 128.0, 128.2, 128.7, 129.6, 130.1, 131.2, 131.3, 133.6, 134.0, 139.2, 139.5, 139.7, 141.2, 142.2, 147.6, 147.7, 162.3; *m*/*z* 396 (100, MH^+^); HRMS (ES): MH^+^, found 396.1045. C_25_H_18_NO_2_S^+^ requires 396.1058.

*Methyl 6,8-bis(4-fluorophenyl)thieno[3,2-*c*]quinoline-2-carboxylate* (**3b**). Solid (0.34 g, 65%), mp. 272–274 °C (toluene); ν_max_ (ATR) 804, 1098, 1159, 1228, 1375, 1487, 1513, 1604, 1666, 1723 cm^−1^; δ_H_ (300 MHz, DMSO-*d*_6_) 3.94 (3H, s, CH_3_), 7.34 (2H, t, *J* 8.7 Hz, 2'',6''-H), 7.38 (2H, t, *J* 8.7 Hz, 2',6'-H -H), 7.81 (2H, t, *J* 8.7 Hz, 3'',5''-H), 8.06 (2H, t, *J* 8.7 Hz, 3',5'-H), 8.08 (1H, d, *J* 1.8 Hz, 7-H), 8.46 (1H, s, 3-H), 8.49 (1H, d, *J* 1.8 Hz, 9-H), 9.33 (1H, s, 4-H); *m*/*z* 432 (100, MH^+^); HRMS (ES): MH^+^, found 432.0870. C_25_H_16_NO_2_SF_2_^+^ requires 432.0870.

*Methyl 6,8-bis(4-methoxyphenyl)thieno[3,2-*c*]quinoline-2-carboxylate* (**3c**). Solid (0.38 g, 65%), mp. 140–142 °C (toluene); ν_max_ (ATR) 696, 814, 945, 1065, 1509, 1644, 1724 cm^−1^; δ_H_ (300 MHz, DMSO-*d*_6_) 3.84 (3H, s, -OCH_3_), 3.85 (3H, s, -OCH_3_), 3.95 (3H, s, -C(O)OCH_3_), 7.06 (2H, d, *J* 8.7 Hz, 3'',5''-H), 7.10 (2H, d, *J* 8.7 Hz, 3',5'-H), 7.71 (2H, d, *J* 8.7 Hz, 2'',6''-H),7.93 (2H, d, *J* 8.7 Hz, 2',6'-H), 8.04 (1H, d, *J* 1.8 Hz, 7-H), 8.39 (1H, d, *J* 1.8 Hz, 9-H), 8.47 (1H, s, 3-H), 9.32 (1H, s, 4-H); δ_C_ (75 MHz, DMSO-*d*_6_) 53.5, 55.7, 55.8, 113.8, 115.1, 119.7, 124.5, 129.3, 129.7, 131.4, 131.6, 132.0, 132.6, 133.7, 134.0, 139.4, 141.0, 141.8, 147.4, 147.8, 159.3, 160.1, 162.4; *m*/*z* 456 (100, MH^+^); HRMS (ES): MH^+^, found 456.1273. C_27_H_22_NO_4_S^+^ requires 456.1270.

*Methyl 6,8-bis(4-fluorophenylethen-1-yl)thieno[3,2-*c*]quinoline-2-carboxylate* (**3d**). Solid (0.58 g, 96%), mp. 208–210 °C (toluene); ν_max_ (ATR) 747, 842, 956, 1058, 1288, 1507, 1550, 1632, 1720 cm^−1^; δ_H_ (300 MHz, DMSO-*d*_6_) 3.90 (3H, s, -CH_3_), 7.25 (2H, t, *J* 8.7 Hz, 2'',6''-H), 8.26 (2H, t, *J* 8.7 Hz, 2',6'-H), 7.37 (1H, d, *J*_trans_ 16.8 Hz, vinyl-H), 7.55 (1H, d, *J*_trans_ 16.8 Hz, vinyl-H), 7.61 (1H, d, *J*_trans_ 16.8 Hz, vinyl-H), 7.70 (2H, t, *J* 8.7 Hz, 3'',5''-H), 7.71 (2H, t, *J* 8.7 Hz, 3',5'-H), 8.14 (1H, d, *J* 1.8 Hz, 7-H). 8.30 (1H, d, *J*_trans_ 16.8 Hz, vinyl-H), 8.37 (1H, s, 3-H), 8.38 (1H, d, *J* 1.8 Hz, 9-H); 9.28 (1H, s, 4-H); δ_C_ (75 MHz, DMSO-*d*_6_) 53.4, 116.2 (d, ^2^*J*_CF_ 21.3 Hz, 2 × C), 121.2, 123.7, 123.9, 124.5, 127.7, 129.0 (d, ^3^*J*_CF_ 8.1 Hz), 129.1(d, ^3^*J*_CF_ 8.0 Hz), 130.1, 131.4, 133.4, 133.8, 133.9 (d, ^4^*J*_CF_ 3.1 Hz), 134.3 (d, ^4^*J*_CF_ 3.2 Hz), 136.6, 141.2, 146.9, 147.4, 162.2, 162.3 (d, ^1^*J*_CF_ 243.6 Hz), 162.4 (d, ^1^*J*_CF_ 243.8 Hz); *m*/*z* 484 (100, MH^+^); HRMS (ES): MH^+^, found 484.1181. C_29_H_20_NO_2_F_2_S^+^ requires 484.1183.

*Methyl 6,8-bis(4-chlorophenylethen-1-yl)thieno[3,2-*c*]quinoline-2-carboxylate* (**3e**). Solid (0.52 g, 81%), mp. 177–178 °C (toluene); ν_max_ (ATR) 750, 620, 1005, 1072, 1088, 1160, 1288, 1479, 1694 cm^−1^; δ_H_ (300 MHz, DMSO-*d*_6_) 3.92 (3H, s, -CH_3_), 7.35–7.47 (6H, m, Ar-H), 7.55 (1H, d, *J*_trans_ 16.8 Hz, vinyl-H), 7.46 (4H, d, *J* 7.5 Hz, 2′,6′-H and 2″,6″-H), 8.08 (1H, d, *J* 1.8 Hz, 7-H), 8.29 (1H, d, *J*_trans_ 16.5 Hz, vinyl-H), 8.31 (1H, s, 3-H), 8.38 (1H, d, *J* 1.8 Hz, 9-H), 9.25 (1H, s, 4-H); *m*/*z* 516 (100, MH^+^); HRMS (ES): MH^+^, found 516.0598. C_29_H_20_NO_2_S^35^Cl_2_^+^ requires 516.0588.

#### 3.1.3. Typical Procedure for the Preparation of Compounds **4a**–**e**

A mixture of **2** (1 equiv.), arylboronic acid (2.5 equiv.), PdCl_2_(PCy_3_)_2_ (10% of **2**) and K_2_CO_3_ (2.5 equiv.) in DMF-ethanol (3:1, v/v; 5 mL/mmol of **2**) in a two-necked flask equipped with a stirrer bar, rubber septum and a condenser was flushed for 20 min with argon gas. A balloon filled with argon gas was then connected to the top of the condenser and the mixture was heated with stirring at 80–90 °C under argon atmosphere for 4 h. The mixture was allowed to cool to room temperature and then poured into an ice-cold water. The resulting precipitate was filtered on a sintered funnel and recrystallized to afford **3**. The following products were prepared in this fashion:

*Ethyl 6,8-diphenylthieno[3,2-*c*]quinoline-2-carboxylate* (**4a**). Solid (0,48 g, 96%), mp. 176–178 °C (toluene); ν_max_ (ATR) 680, 753, 886, 1063, 1280, 1577, 1698, 1748 cm^−1^; δ_H_ (300 MHz, DMSO-*d*_6_) 1.37 (3H, t, *J* 6.9 Hz, -CH_3_), 4.39 (2H, q, *J* 6.9 Hz, -CH_2_-), 7.44–7.58 (6H, m, Ar-H), 7.75 (2H, d, *J* 7.5 Hz, 2'',6''-H), 7.99 (2H, d, *J* 7.5 Hz, 2',6'-H), 8.09 (1H, d, *J* 1.8 Hz, 9-H), 8.46 (1H, s, 3-H), 8.50 (1H, d, *J* 1.8 Hz, 7-H), 9.34 (1H, s, 4-H); δ_C_ (75 MHz, DMSO-*d*_6_) 14.6, 62.3, 121.1, 124.3, 127.9, 128.0, 128.3, 128.8, 129.6, 130.1, 131.2, 131.3, 133.7, 134.4, 139.2, 139.6, 139.7, 141.3, 142.2, 147.8, 147.9, 161.9; *m*/*z* 410 (100, MH^+^); HRMS (ES): MH^+^, found 410.1216. C_26_H_20_NO_2_S^+^ requires 410.1215.

*Ethyl 6,8-bis(4-fluorophenyl)thieno[3,2-*c*]quinoline-2-carboxylate* (**4b**). Solid (0.38 g, 70%), mp. 242–243 °C (toluene); ν_max_ (ATR) 639, 819, 1064, 1230, 1396, 1509, 1635, 1722 cm^−1^; δ_H_ (300 MHz, DMSO-*d*_6_) 1.46 (3H, t, *J* 6.3 Hz, -CH_3_), 4.37 (2H, q, *J* 6.3 Hz, -CH_2_-), 7.32 (2H, t, *J* 8.7 Hz, 2'',6''-H), 7.36 (2H, t, *J* 8.7 Hz, 2',6'-H), 7.78 (2H, t, *J* 8.7 Hz, 3'',5''-H), 8.04 (2H, t, *J* 8.7 Hz, 3',5'-H), 8.05 (1H, d, *J* 1.8 Hz, 9-H), 8.39 (1H, d, *J* 1.8 Hz, 7-H), 8.45 (1H, s, 4-H), 9.30 (1H, s, 4-H); *m*/*z* 446 (100, MH^+^); HRMS (ES): MH^+^, found 446.1024. C_26_H_18_NO_2_F_2_S^+^ requires 446.1026.

*Ethyl 6,8-bis(4-chlorophenyl)thieno[3,2-*c*]quinoline-2-carboxylate* (**4c**). Solid (0.35 g, 71%), mp. 204–206 °C (toluene); ν_max_ (ATR) 750, 820, 1072, 1088, 1160, 1288, 1478, 1694 cm^−1^; δ_H_ (300 MHz, DMSO-*d*_6_) 1.32 (3H, t, *J* 6.3 Hz, -CH_3_), 4.32 (2H, q, *J* 6.3 Hz, -CH_2_-), 7.48–7.53 (4H, m, 3',5'-H, 3'',5''-H), 7.70 (2H, d, *J* 7.8 Hz, 2'',6''-H), 7.93 (2H, d, *J* 7.8 Hz, 2',6'-H), 7.98 (1H, s, 3-H), 8.27 (1H, d, *J* 1.8 Hz, 7-H), 8.36 (1H, d, *J* 1.8 Hz, 9-H), 9.21 (1H, s, 4-H); δ_C_ (75 MHz, DMSO-*d*_6_) 14.6, 52.2, 121.5, 123.9, 125.3, 127.5, 128.6, 128.8, 129.3, 129.7, 131.1, 132.7, 132.8, 133.7, 134.1, 136.3 (2 × C), 136.7, 141.2, 147.0, 147.3, 161.7; *m*/*z* 478 (100, MH^+^); HRMS (ES): MH^+^, found 478.0869. C_26_H_18_NO_2_S^35^Cl_2_^+^ requires 478.0870.

*Ethyl 6,8-bis(4-methoxyphenyl)thieno[3,2-*c*]quinoline-2-carboxylate* (**4d**). Solid (0.37 g, 62%), mp. 131–132 °C (toluene); ν_max_ (ATR) 749, 931, 1065, 1238, 1403, 1509, 1633, 1715 cm^−1^; δ_H_ (300 MHz, DMSO-*d*_6_) 1.36 (3H, t, *J* 6.3 Hz, -CH_3_), 3.83 (3H, s, -OCH_3_), 3.84 (3H, s, -OCH_3_), 4.37 (2H, q, *J* 6.3 Hz, -CH_2_-), 7.05 (2H, d, *J* 8.4 Hz, 3'',5''-H), 7.08 (2H, d, *J* 8.4 Hz, 3',5'-H), 7.68 (2H, d, *J* 8.4 Hz, 2'',6''-H), 7.90 (2H, d, *J* 8.4 Hz, 2',6'-H), 7.99 (1H, d, *J* 1.8 Hz, 7-H), 8.32 (1H, d, *J* 1.8 Hz, 9-H), 8.38 (1H, s, 3-H), 9.26 (1H, s, 4-H); δ_C_ (75 MHz, DMSO-*d*_6_) 14.6, 55.6, 55.7, 62.2, 113.7, 114.9, 119.5, 124.3, 128.7, 129.1, 129.4, 131.1, 131.9, 132.5, 133.5, 134.1, 139.1, 140.9, 141.6, 147.1, 147.5, 159.2, 160.0, 161.8; *m*/*z* 470 (100, MH^+^); HRMS (ES): MH^+^, found 470.1424. C_28_H_24_NO_4_S^+^ requires 470.1426.

*Ethyl 6,8-bis(4-chlorophenylethen-1-yl)thieno[3,2-*c*]quinoline-2-carboxylate* (**4e**). Solid (0.38 g, 67%), mp. 168–170 °C (toluene); ν_max_ (ATR) 572, 746, 1056, 1245, 1591, 1626, 1712, 1788 cm^−1^; δ_H_ (300 MHz, DMSO-*d*_6_) 1.36 (3H, t, *J* 6.9 Hz, -CH_3_), 4.36 (2H, q, *J* 6.9 Hz, -CH_2_-), 7.42–7.72 (12H, m, Ar-H, 7-H and vinyl-H), 8.21 (1H, d, *J* 1.8 Hz, 9-H), 8.37 (1H, s, 3-H), 8.38 (1H, d, *J*_trans_ 16.5 Hz, vinyl-H), 9.32 (1H, s, 4-H); δ_C_ (75 MHz, DMSO-*d*_6_) 14.6, 62.3, 121.5, 123.8, 125.3, 127.6, 128.5, 128.6, 128.8, 129.3, 129.7, 130.5, 131.2, 132.3, 132.4, 132.7, 132.8, 133.8, 134.2, 136.3, 136.6, 136.7, 141.2, 147.0, 147.4, 161.8; *m*/*z* 530 (100, MH^+^); HRMS (ES): MH^+^, found 530.0740. C_30_H_22_NO_2_S^35^Cl_2_^+^ requires 530.0748.

### 3.2. Cell Culture and Conditions

Stock solutions of all thienoquinoline derivatives (2 × 10^−2^ μg/mL) were made with DMSO and stored at room temperature. Human breast adenocarcinoma cells MCF-7 were maintained in Dulbecco’s modified eagle’s medium (DMEM) (Sigma-Aldrich, St. Louis, MO, USA), supplemented with 10% heat inactivated fetal calf serum (Invitrogen, Carlsbad, CA, USA) and 1% Penicillin-streptomycin (Whitehead Scientific, Brackenfell, South Africa). The cells were maintained at a temperature of 37 °C in a humidified atmosphere containing 5% CO_2_.

#### 3.2.1. Tetrazolium-Based Cytotoxicity Analysis

The cytotoxic effects of the compounds were determined using the tetrazolium based colorimetric cell viability assay (methylthiazolyltetrazolium (MTT) assay) against MCF-7 cells using the method described by Mosmann [28]. Cells were seeded into 96-well plates (Greiner, Frickenhausen, Germany) at a density of 5000 cells per well for 24 h. The seeding density depended on the growth characteristics of the cells and was chosen to avoid a 100% confluency of untreated cells. After 24 h, the cells were treated with varying concentrations of test compound (0.01–10 µg/mL) or nocodazole (0.01–10 µg/mL) for 48 h. The cells were rinsed with PBS, and 20 μL of methylthiazolyltetrazolium (MTT) solution (5.00 mg/mL) were added to each well and incubated for 4 h. Incubation with MTT was terminated after 4 h by adding 100 μL of a mixture of 2-propanol: 1 M HCl (50 mL:165 μL) to each well. Then, the plates were kept at 37 °C for 1 h and the absorbance was measured at 570 nm (test wavelength) and 690 nm (reference wavelength) using the ELx 800 Universal Microplate Reader (Bio-Tek Instruments Inc., Winooski, VT, USA). Each dilution/concentration of the test sample was tested in quadruplicate. Studies were undertaken in triplicate. The percentage cell viability was calculated using the formula below:

(1) $$\%\;\text{cell viability}=\frac{\text{Mean Absorbance of sample}}{\text{Mean Absorbance of control}}\times 100$$

The LC_50_ values (lethal concentration at which 50% of the cells are killed) were as the concentration of the test sample that resulted in 50% reduction of absorbance compared to untreated cells. The intensity of the MTT formazan produced by living metabolically active cells as determined by the correlating absorbance reading is directly proportional to the number of live cells present [28].

#### 3.2.2. xCelligency Real Time Cell Analysis Proliferation Assay

The xCELLigence system (Roche Applied Science, Ranburg, South Africa) was employed in this study. A volume of 100 µL serum DMEM was added to each well of the E-plate (Roche Diagnostics) and the plates were allowed to equilibrate at room temperature. The plates were then calibrated on the xCELLigence system. Thereafter, 6000 cells were added to each well and the plates were incubated at room temperature to allow the cells to settle. The plates were then placed in the xCELLigence system. After 21 h, the cells were treated with varying concentrations of test compound (0.01–10 µg/mL) or nocodazole (0.01–10 µg/mL). Control cells were treated with DMSO. The cells were monitored with an xCELLigence RTCA instrument for 56 h. The cell index (CI) values were recorded by the instrument analyzer and analysis was performed with the supplied RTCA software (version 1.2.1).

## 4. Conclusions

The 6,8-dibromothieno[3,2-*c*]quinoline-2-carboxylate scaffold enabled transformation through palladium catalyzed Suzuki-Miyaura cross-coupling with arylboronic acids to afford novel polysubstituted thieno[3,2-*c*]quinoline-2-carboxylates that would be difficult to prepare otherwise. The cytotoxic properties of the thieno[3,2-*c*]quinolines prepared in this investigation were found to be concentration-dependent. The level of cytotoxicity seems to depend on the electronic effects and lipophilicity of the substituent on the *para* position of the 6- and 8-phenyl rings. Although the mechanism of action of these thienoquinolines is not known to us at this time, the structure-activity relationship analysis provides a guideline for the design of new derivatives with increased activity. This can be achieved by varying substitution pattern on the aryl substituents or by replacement of the aryl groups with heteroaryl rings (via Suzuki, Negishi, or Stille cross-coupling reactions) or heteroatom-containing groups (Buchwald-Hartwig cross-coupling) to lead to more potent derivatives. 
